# Modeling dynamics and alternative treatment strategies in acute promyelocytic leukemia

**DOI:** 10.1371/journal.pone.0221011

**Published:** 2019-08-15

**Authors:** Gerson Hiroshi Yoshinari, Artur César Fassoni, Luis Fernando Mello, Eduardo M. Rego

**Affiliations:** 1 Faculdade de Medicina de Ribeirão Preto, Universidade de São Paulo, Ribeirão Preto, SP, Brazil; 2 Instituto de Matemática e Computação, Universidade Federal de Itajubá, Itajubá, MG, Brazil; 3 Faculdade de Medicina da Universidade de São Paulo and Center for Cell Based Therapy, University of São Paulo, São Paulo, SP, Brazil; 4 Divisão de Hematologia, LIM31, Faculdade de Medicina, Universidade de São Paulo, São Paulo, SP, Brazil; University of California Irvine, UNITED STATES

## Abstract

Acute Promyelocytic Leukemia (APL) is a rare and potentially lethal condition in which risk-based therapy often leads to better outcomes. Because of its rarity and relatively high overall survival rate, prospective randomized trials to investigate alternative APL treatment schedules are challenging. Mathematical models may provide useful information in this regard. We collected clinical data from 38 patients treated for APL under the International Consortium on Acute Leukemia (ICAL) protocol and laboratory data during induction therapy. We propose a mathematical model that represents the dynamics of leukocytes in peripheral blood and the effect of ICAL treatment on the disease’s dynamics. We observe that our cohort presents demographic characteristics and clinical outcomes similar to previous clinical trials on APL. Over a follow-up period of 41.8 months, the relapse-free survival and overall survival at two years are both found to be 78.7%. For two selected patients, the model produces a good fit to the clinical data. Information such as the response to treatment and risk of relapse can be derived from the model, and this may assist in clinical practice and the design of clinical trials.

## Introduction

Acute Promyelocytic Leukemia (APL) is a subset of Acute Myeloid Leukemia (AML) that is clinically characterized by gene rearrangements involving the *Retinoic Acid Receptor Alpha* (RARα) and by the infiltration of bone marrow and/or blood by leukemic cells that resemble promyelocytes. It is an unusual condition: its bimodal incidence reaches 3.7 per 100,000 persons, corresponding to 10% of all AML cases in Caucasians from the US, UK, and Scandinavia [1] and approximately 20% in patients with Latino ancestry in the US and Latin America [2]. In the past, APL was one of the most lethal forms of AML, but improvements in therapeutics have rendered it a potentially curable disease [3,4]. Well-established treatment protocols based on the use of all-trans retinoic acid (ATRA), a vitamin A derivative, and anthracyclines have resulted in long-term overall survival rates above 70% [4–6]. Association with arsenic trioxide (ATO) has led to high complete remission rates and long disease-free survival in European [7,8] and Australian [9] trials, and has become the standard of care in several developed countries. Nevertheless, the cost of ATO is a major hurdle for developing countries. On the initiative of the International Members Committee (IMC) of the American Society of Hematology (ASH), the International Consortium on Acute Leukemia (ICAL) assembled a network of institutions in Latin America and developed a protocol of diagnosis, treatment, and supportive care adapted to local resources [3,4]. The results from this ICAL study were similar to those reported in developed countries with protocols based on ATRA and anthracyclines, and provided substantial clinical data [4].

Despite the improvement in overall survival (OS) and relapse-free survival (RFS) provided by the ATRA and anthracyclines regimen, the associated toxicity must be taken into consideration. For example, anthracyclines have a well-known dose-related cardiac and myelosuppressive toxicity, and ATRA may lead to the “differentiation syndrome” [10–12]. Additionally, cytarabine is used for high-risk patients in protocols based on the PETHEMA/GIMEMA trials, and this is associated with myelosuppression. Thus, it is imperative to establish a distinction between patients who have a higher risk of relapse, and would benefit from a more intense drug regimen, and patients with a lower risk of relapse, for whom less intense protocols would be sufficient and lead to fewer side effects. Currently, a risk-adapted treatment protocol that takes into consideration the global count of leukocytes and platelets in peripheral blood is used to determine the risk of relapse, and this has demonstrated improved results in comparison to a non-adapted protocol [4,6,13,14]. However, there is no clear consensus on the establishment of this risk [15–17].

Another question arising from the good response presented by some patients regards treatment optimization, i.e., how to optimize the patient’s treatment by maximizing the response and minimizing the toxicity. Mathematical models may be used to suggest relevant strategies in APL treatment, as there are no large patient databases that can be used as a reliable data mining tool for prognosis. Thus, modeling is essential to determine the intensity of the treatment regimen.

In the present study, clinical data from a set of patients treated under the ICAL protocol were analyzed. A mathematical model based on an ecological paradigm is proposed to describe the first 30 days of the induction stage of the ICAL treatment protocol. Long-term analysis of the model and its clinical interpretation will be discussed. The model is fitted to individual patient time courses, allowing alternative treatment schedules based on the model simulations to be investigated. Finally, future directions of study on this topic are discussed.

## Methods

### Clinical data from patients included in ICAL protocol

The development and study design of the ICAL protocol have been reported elsewhere [4]. Data from all patients with APL treated under the ICAL protocol in the *Hospital das Clínicas de Ribeirão Preto* (*HCRP*, São Paulo, Brazil) were retrieved. The extraction and usage of these data were previously authorized by the local and national Institutional Review Board (IRB) under protocol no. 66129717.1.0000.5440. No patient consent was required because only anonymous data were retrieved. Demographic data (gender, age, weight at diagnosis), clinical outcomes of APL (remission, bleeding episodes, relapse, death), treatment toxicity events (cardiologic and others), and risk of relapse were obtained. Relevant laboratory data, such as hemoglobin, hematocrit, global and differential white globe counts, platelets, coagulation markers, creatinine, and albumin for 1–7, 14, 21, and 28 days after the diagnosis of APL were also extracted.

We used MedCalc v.12.5.0 to conduct the statistical analysis and Mathematica v.11.2 for the model simulations.

### The proposed APL model

In a seminal paper, Clarkson proposed the interpretation of AML as a two-population disease of normal and leukemic cells [18]. Our assumptions of the behavior of these populations are based on previous models [19,20], but certain characteristics are proposed to comprehend the competition between them:

1. The populations of normal white blood cells in the peripheral blood from a single normal cell population and the promyelocytes and myeloblasts form a single leukemic cell population. The dynamics in the peripheral blood compartment and bone marrow compartment are assumed to be equivalent, which means that the population behavior in the peripheral blood represents the population behavior in the bone marrow. This simplification is intended to reduce the model complexity, and is plausible because the clinical outcome in APL patients is assessed by peripheral blood analysis [21].
2. For APL, leukemic cells are interpreted as an immature form of the normal white blood cells which, under ATRA stimuli, can mature into the latter population.
3. Normal cell growth follows a constant flow. This is because the production of new normal cells does not depend directly on the total number of living normal cells, but is an intrinsic property of the tissue in order to maintain homeostasis.
4. Leukemic cells are in the proliferative state and maintain their growth program, independent of the tissue’s structure. Thus, in this case, density-dependent growth is considered [22]. Logistic growth is chosen for its simplicity.
5. Leukemic cells exert a negative and inhibitory effect on the growth and development of the normal cells through interactive mechanisms such as competition for oxygen and nutrients, expression of inhibitory markers, and so on. Conversely, normal cells exert a negative effect on leukemic cells.
6. A certain proportion of normal and leukemic cells are destroyed under inhibition from the antagonist population and through cell death.
7. As there is no solid tumor burden, the spatial, angiogenic, hypoxemic, and diffusion aspects are not considered.

Fassoni and Yang proposed a set of ordinary differential equations (ODEs) that consider these conditions [23]. Let *N*(*t*) be a function depending on time *t* that represents the number of normal leukocytes and *A*(*t*) be a function that represents the number of leukemic cells over time. We present a pair of ODEs describing the assumptions stated above. The dependence on time can be omitted because the set of ODEs is autonomous:

$$\frac{dN}{dt}=r_{N}-\mu_{N}N-\beta_{1}NA,$$ (Eq 1)

$$\frac{dA}{dt}=r_{A}A\left(1-\frac{A}{K_{A}}\right)-\beta_{2}NA-\mu_{A}A.$$ (Eq 2)

The functions *N*(*t*) and *A*(*t*) take non-negative real values. The parameter *r*_*N*_ represents the total constant reproduction of normal cells and *μ*_*N*_ is their natural mortality. The homeostatic state of the normal leukocyte population is given by *r*_*N*_/*μ*_*N*_. The parameter *r*_*A*_ represents the per capita growth rate of population *A*, *K*_*A*_ is the carrying capacity, and *μ*_*A*_ represents the natural mortality rate of the population. *β*_1_ represents, in a general sense, the negative and inhibitory effect of leukemic cells over the normal leukocytes, and *β*_2_ denotes the negative and inhibitory effect of normal leukocytes over the population of leukemic cells.

Eqs (1) and (2) describe the dynamics between APL and normal leukocytes in the untreated case. We shall extend this model to incorporate the first 30 days of induction therapy (see “Introducing treatment effect in the proposed APL model”).

## Results

### Clinical data

Table 1 presents the clinical data retrieved from 38 patients with APL treated at HCRP under the International Consortium on Acute Promyelocytic Leukemia (IC-APL) [4] protocol of the ICAL from 2007–2017. The median time until hematologic remission was 38 days. Our data cover the first 30 days of the induction protocol. The median follow-up was 41.8 months.

**Table 1:** Demographic and clinical data from ICAL patients at HCRP.

Variable	Value (±SD)	Percentage
Mean age (in years)	38.4±15.2	NA
Weight (in kilograms)	73.2±12.3	NA
Male	20	52.6%
Female	18	47.4%
Bleeding episode	14	36.8%
Treatment toxicity	10	26.3%
Cardiac toxicity	4	10.5%
Febrile neutropenia	4	10.5%
Other	2	5.3%
Deaths	9	23.7%
Deaths pre-remission	5	13.1%
Relapse	6	15.7%
Relapse during Maintenance therapy	4	10.5%
Relapse risk–PETHEMA/GIMEMA criteria [14]		
High-risk	9	23.7%
Intermediate-risk	26	68.4%
Low-risk	3	7.9%

**Fig 1:**
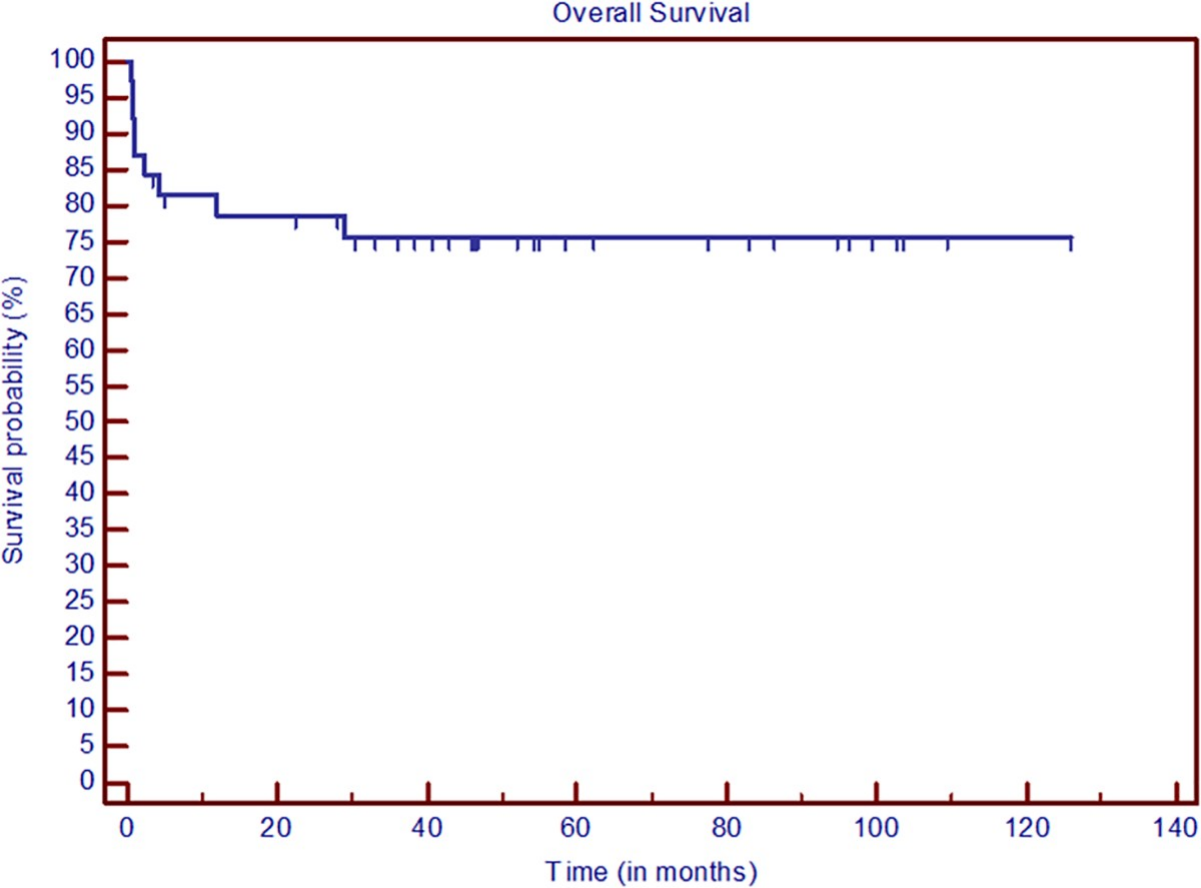
Kaplan–Meier OS curves.

**Fig 2:**
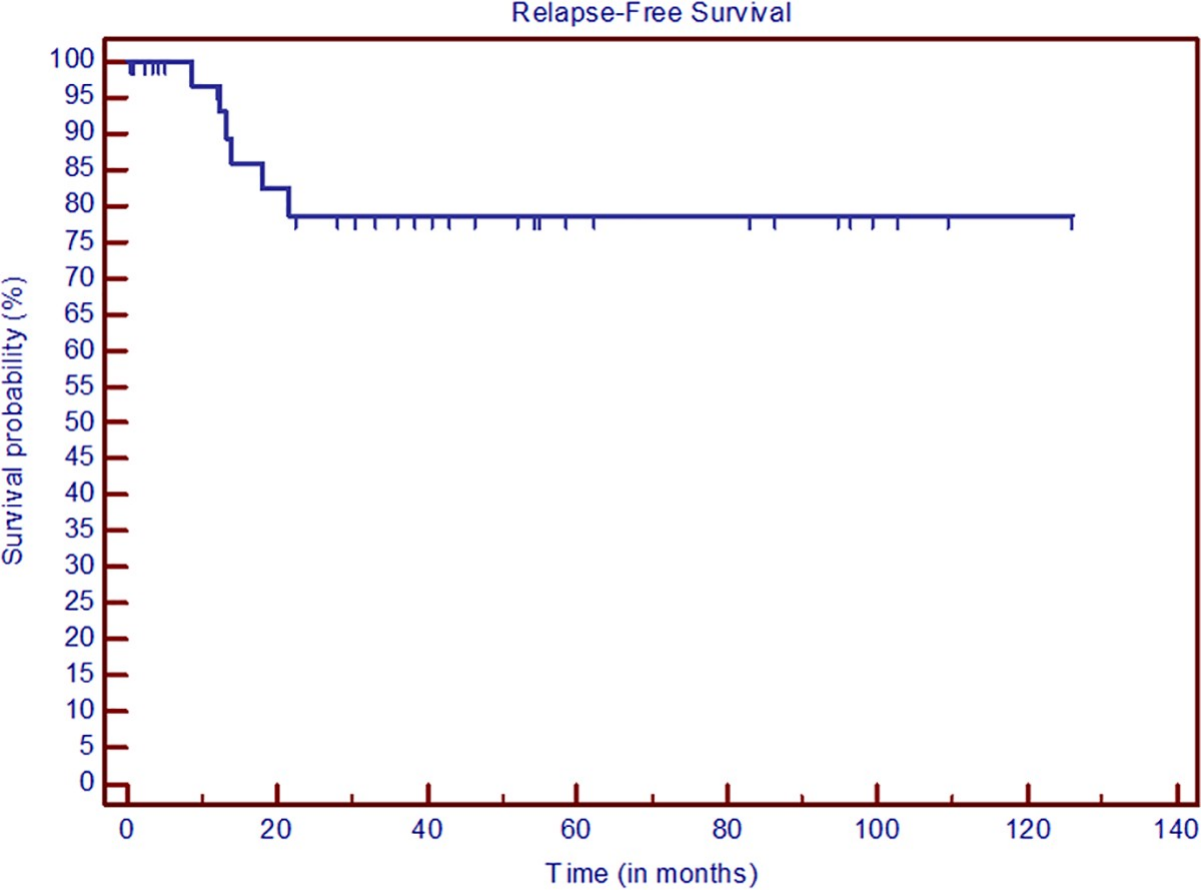
Kaplan–Meier RFS curves.

**Fig 3:**
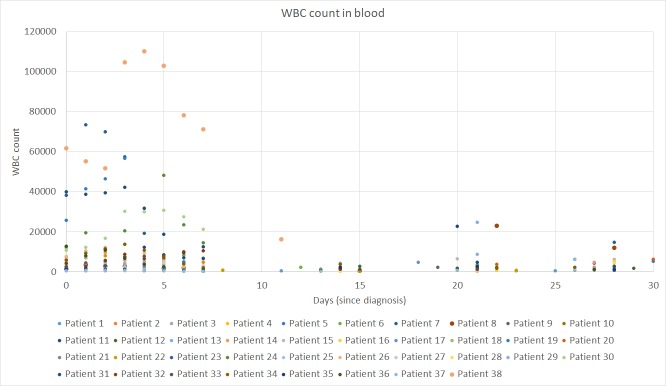
WBC count over the first 30 days of induction therapy.

The Kaplan–Meier survival curves for OS and RFS are presented in Figs 1 and 2. The white blood cell (WBC) count in peripheral blood over the first 30 days of induction therapy is shown in Fig 3.

Cardiologic toxicity was found in four patients during the follow-up, where two patients presented transient heart failure with a left ventricular ejection fraction (LVEF) heading back to baseline. Three patients were asymptomatic at their latest medical checks. Only one patient presented dyspnea at rest, although their baseline LVEF was 38% and 39% at the most recent examination.

### Asymptotic behavior of the solutions as a predictor of clinical outcome

**Fig 4:**
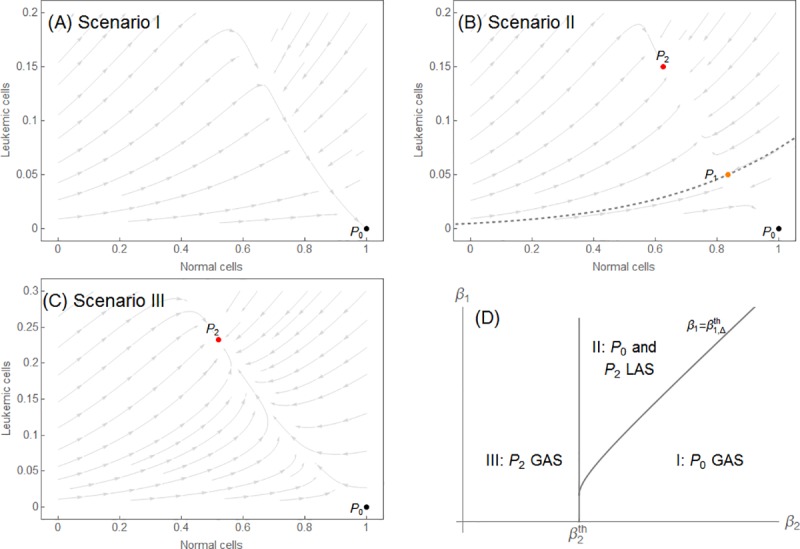
Phase portraits of subsystem N × A for every scenario described. In (A), Scenario I, *P*_0_ is globally asymptotically stable (GAS). In (B), Scenario II, the stable curve of *P*_1_ divides the phase plane into basins of attraction for *P*_0_ and *P*_2_, which are locally asymptotically stable (LAS). In (C), Scenario III presents *P*_2_ as GAS for initial conditions *A*(0)>0. In (D), we present the general behavior of the parameter space for *β*_1_ and *β*_2_.

The equilibrium points (or steady states) of the ODEs can be interpreted as the long-term clinical outcome of the patient (asymptotic behavior). System (1) has three different steady states: *P*_0_ = (*r*_*N*_/*μ*_*N*_,0), *P*_1_ = (*N*_1_,*A*_1_), and *P*_2_ = (*N*_2_,*A*_2_), where *A*_1_ and *A*_2_ are roots of a second-degree polynomial and *N*_*i*_ = *r*_*N*_/(*μ*_*N*_+*b*_1_*A_*i*_). Equilibrium *P*_0_ corresponds to the extinction of leukemic cells, whereas *P*_1_ and *P*_2_ correspond to the coexistence of normal and leukemic cells. A complete mathematical analysis of the existence, positiveness, and stability of these steady states has previously been conducted in a more general context [23]. Here, we briefly present some results that can be interpreted in the context of APL treatment. System (1) presents three distinct qualitative scenarios in its phase portrait (Fig 4). Depending on the parameter values, one of three scenarios emerges:

**Scenario I**: there is only one equilibrium point *P*_0_, and it is globally asymptotically stable. Leukemia is eliminated, whatever the initial conditions (Fig 4A).

**Scenario II**: three equilibrium points coexist in the first quadrant. *P*_0_ and *P*_2_ are locally asymptotically stable; *P*_1_ is a saddle point, and its stable curve is the separatrix between the basins of attraction of *P*_0_ and *P*_2_. Leukemia may develop or be eliminated. The outcome depends on the initial condition (Fig 4B).

**Scenario III**: there are two equilibrium points, and *P*_2_ is almost globally asymptotically stable. Leukemia develops, whatever the initial condition (Fig 4C).

The conditions that lead to each scenario can be described in terms of the parameters *β*_1_ and *β*_2_, and particularly the thresholds $\beta_{2}^{th}$ and $\beta_{1,\Delta }^{th}$. The exact conditions are as follows (see also Fig 4D): $\beta_{2}<\beta_{2}^{th}$ leads to Scenario III; $\beta_{2}>\beta_{2}^{th}$ and $\beta_{1}>\beta_{1,\Delta }^{th}$ leads to Scenario II, whereas $\beta_{2}>\beta_{2}^{th}$ and $\beta_{1}<\beta_{1,\Delta }^{th}$ leads to Scenario I. The expressions for the thresholds are $\beta_{2}^{th}={(\mu }_{N}/r_{N})l_{A}$,

where *l*_*A*_ = *r*_*A*_−*μ*_*A*_, and $\beta_{1,\Delta }^{th}=\beta_{1}^{th}+2\eta +2\sqrt{\eta (\beta_{1}^{th}+\eta )}$, where $\eta =r_{A}r_{N}(\beta_{2}-\beta_{2}^{th})/(K_{A}l_{A}^{2}).$ A complete mathematical analysis of system (1) and the derivation of these conditions were given in [23].

The initial conditions of the disease are given by aleatory events (representing mutations, epigenetic modifications on cancer stem cells, and so on) that “initialize” the leukemic cell population with its intrinsic characteristics; these events are not the focus of this paper. The equilibria may be interpreted as the long-term outcomes for the patient depending on the cell population, where *P*_0_ and *P*_2_ represent “disease-free” and “disease” outcomes, respectively. Thus, Scenario I represents a disease that has not become clinically relevant. Scenario II admits two different outcomes: disease or no disease, depending on the initial conditions of the populations. If it is contained in the basin of attraction of *P*_0_, then the disease will not become clinically relevant. However, if the initial condition lies within the basin of attraction of *P*_2_, then the patient will develop APL. Scenario III only allows a long-term disease outcome, in which the patient will evolve into a persistent APL condition regardless of the initial conditions. More details about how the parameter values determine each scenario can be found in [23].

### Introducing the treatment effect in the proposed APL model

The ICAL treatment protocol is based on risk-adapted regimens of cytotoxic chemotherapy and ATRA. Briefly, the protocol comprehends the induction, consolidation, and maintenance therapies [6]. In the induction therapy, the main drugs used are ATRA and daunorubicin. ATRA transforms immature forms into mature forms of the myeloid lineage. An *in vitro* study shows that, above a particular concentration, over 90% of promyelocytes are converted into mature cells in five days [24]. These results were confirmed *in vivo* [5,25]. Daunorubicin is a cytotoxic agent (class of anthracyclines) used in APL management [26]. Its pharmacology depends on the drug delivery system (DDS). For example, Dorlhiac-Llacer *et al*. studied the *in vitro* behavior of daunorubicin associated with low-density lipoprotein emulsion (LDE). They found a much higher cytotoxic activity in leukemic cells for LDE daunorubicin in comparison to no-LDE presentation [27]. As there is no specification of the DDS for daunorubicin in the ICAL protocol, we will assume that no-LDE was administered. Therefore, daunorubicin indiscriminately kills leukemic and normal cells, depending on the dose, drug biological half-life, and administration interval.

We can now introduce induction therapy into the APL model. The ATRA effect causes leukemic cells to mature into normal cells at a constant rate *β*_3_. In the induction therapy, we assume a continuous ATRA administration from day *t* = 0 until day *t* = *t*_*F*_ = 30, which approximates a daily ATRA intake over 30 days. Regarding chemotherapy, the daunorubicin effect instantaneously kills the same proportion of leukemic and normal cells and preserves some of its cytotoxic effect according to its half-life time (26.7 h), as described by a decay rate *τ* [28]. Four daunorubicin doses of *ρ* = 60 mg/m^2^ are given at days *t*_*i*_ = 2, 4, 6, and 8 during induction therapy. We assume a superficial body index of 1.82 m^2^.

Hence, the APL model considering induction therapy may be presented as:

$$\frac{dN}{dt}=r_{N}-\mu_{N}N-\beta_{1}NA+\beta_{3}A(1-u(t-t_{F}))-\gamma DN,$$ (Eq 3)

$$\frac{dD}{dt}=\sum_{i=1}^{N}\rho_{i}\delta (t-t_{i})-\tau D,$$ (Eq 4)

$$\frac{dA}{dt}=r_{A}A\left(1-\frac{A}{K_{A}}\right)-\beta_{2}NA-\mu_{A}A-\beta_{3}A(1-u(t-t_{F}))-\gamma DA,$$ (Eq 5)

where the on/off switch for the ATRA treatment is described by a unit step function: *u*(*t*−*t*_*F*_) = 0 if *0*<*t*<*t*_*F*_ and *u*(*t*−*t*_*F*_) = 1 if *t*>*t*_*F*_; *D*(*t*) is the chemotherapy concentration, and the intake (*ρ*_*i*_ mg at time *t*_*i*_) is described by Dirac’s delta function *δ*(*t*−*t*_*i*_).

The induction treatment does not affect the asymptotic behavior of the solutions, because *β*_3_(1−*u*(*t*−*t*_*F*_)) = 0 and *D*(*t*)→0 after the induction therapy (*t*>*t*_*F*_), so the solutions of Eqs (3–5) approach the solutions of Eqs 1 and 2. In this context, the role of the induction treatment is to move the system state (state variables *N*(*t*) and *A*(*t*)) to a favorable region, corresponding to a decrease in the number of leukemic cells and an increase in the normal cells.

In Scenario II, effective treatment would move the system state from the basin of attraction of *P*_2_ to the basin of attraction of *P*_0_. As a clinical interpretation, there would be patients for whom the induction therapy and a less aggressive consolidation therapy would be sufficient to move from a disease to a disease-free scenario. Nevertheless, we must keep in mind that the size and shape of the basin of attraction of *P*_2_ depend on various parameters. Therefore, for each patient, there is a unique level of difficulty in reaching the remission basin of attraction, i.e., of moving the system from that basin. There are methods to estimate this difficulty, but they are beyond the objective of this study [29].

In Scenario III, relapse is unavoidable unless the treatment qualitatively modifies the phase portrait. That can be achieved by other modalities of treatment (e.g., autologous transplants). These situations are not addressed in this paper.

The information provided by the different qualitative phase portraits can be used to estimate the risk of relapse for a patient with APL after the treatment regimens (induction, consolidation, and maintenance). Depending on the patient’s parameter values, one can predict the scenario in which the system is situated. Scenario III can be interpreted as a high risk of relapse, because if no structural change in the system is made, the population of leukemic cells will invariably rise again, producing a relapse scenario. Scenario II can be interpreted as a low-to-intermediate risk scenario, depending on which basin of attraction the initial condition (modified by the treatment) leads to. Scenario I is not considered here, as in real medical practice such patients would not even be diagnosed with APL.

### Fitting the model simulations to the clinical data

To fit the model to the individual time courses of patients within our cohort, we initially considered some of the model parameters to be fixed for all patients. These assumptions are supported by the literature [30,19,27], and the assumed values are presented in Table 2. The rationale for such parameter values is as follows. The normal cell death rate, leukemic proliferation rate, and leukemic cell death rate were chosen based on the mean times for each event to happen in a 70 kg patient. The chemotherapy half-life was established for daunorubicin as previously discussed. The adequacy of the various units was checked.

**Table 2:** Fixed parameter values.

Parameter	Biological interpretation	Value
*μ*_*N*_	Normal cell death rate (day^-1^)	2.88
*r*_*A*_	Leukemic cell proliferation rate (day^-1^)	3.8808
*μ*_*A*_	Leukemic cell death rate (day^-1^)	0.6648
*K*_*A*_	Leukemic cell carrying capacity (cells/mm^3^)	25000
*τ*	log_e_(2)/chemotherapy half-life (hours^-1^)	0.623054
*ρ*_*i*_	Chemotherapy dose (mg/m^2^)	60
*t*_*i*_	Days for chemotherapy dose	2, 4, 6, 8

For each of the other parameters, we chose biologically reasonable intervals. The number of normal leukocytes in the absence of leukemia ranges from 1000–8000 cells/mm^3^ [30]. In the model, this value is given by $\frac{r_{N}}{\mu_{N}}$. Therefore, we allowed *r*_*N*_ to vary in the interval [1000*μ*_*N*_, 8000*μ*_*N*_]. Parameters *β*_1_ and *β*_2_ measure the negative effect of one cell population on the other, and are hard to measure biologically or infer from data in the literature. The same holds for parameters *β*_3_ and *γ*, which measure the effect of ATRA and chemotherapy, respectively, on leukocytes. Through an initial analysis of the simulations, we found reasonable intervals (where the model reproduced expected results) for each of these parameters, with the resulting model behavior ranging from very aggressive to non-aggressive leukemias. Therefore, we took these intervals as the initial range for these parameters (see Table 3). We then generated *K* = 10,000 tuples of random values *X* = (*r*_*n*_,*β*_1_,*β*_2_,*β*_3_,*γ*) by sampling from a uniform distribution in those intervals. For every tuple *X*, we calculated the quadratic error *E(X)* between the model solution and the corresponding laboratory data, defined as:

$$E(X)=\sum_{j=1}^{n_{1}}{\left(N(t_{j})+A(t_{j})-L_{j}\right)}^{2}+\sum_{k=1}^{n_{2}}{\left(A(t_{k})-I_{k}\right)}^{2},$$ (Eq 6)

where *n*_1_, *n*_2_, correspond to the total laboratory measures of total leukocytes *L*_*j*_ and immature leukocytes *I*_*k*_, respectively, and *t*_*j*_, *t*_*k*_ denote the day the laboratory data were measured. *N*(*t*_*j*_)+*A*(*t*_*j*_) and *A*(*t*_*k*_) represent the model solution for the given quantities in the considered time. From the initial 10,000 tuples, we selected the 100 that produced the smallest values of *E(X)*. We then used each of these 100 tuples as the initial condition for a maximum descent optimization method that minimizes *E*(*X*). From this procedure, we obtained 100 tuples in the parameter space corresponding to local minima for *E(X)* (see S1 and S2 Figs). Among these final 100 tuples, we identified that which produced the smallest *E(X)*, excluding those corresponding to Scenario I (clinically irrelevant) [31].

**Table 3:** Intervals for non-fixed parameters and estimated values for patients #18 and #22.

Parameter	Biological interpretation	Interval	Value for Patient #18	Value for Patient #22
*r*_*N*_	Normal cell production rate (cells/(mm^3^ x day))	[2880, 23040]	2193.2	1071.7
*β*_1_	Leukemic cell competition rate ((cells/mm^3^)^-1^day^-1^)	[0, 0.03]	0.001511	0.006588
*β*_2_	Normal cell competition rate ((cells/mm^3^)^-1^day^-1^)	[0, 0.005]	0.003569	0.003259
*β*_3_	Maturation rate of leukemic cells due to ATRA (day^-1^)	[0, 1]	0.289005	0.173926
*γ*	Chemotherapy effect on normal and leukemic cells (mg^-1^day^-1^)	[0, 0.2]	0.093906	0.034529

**Fig 5:**
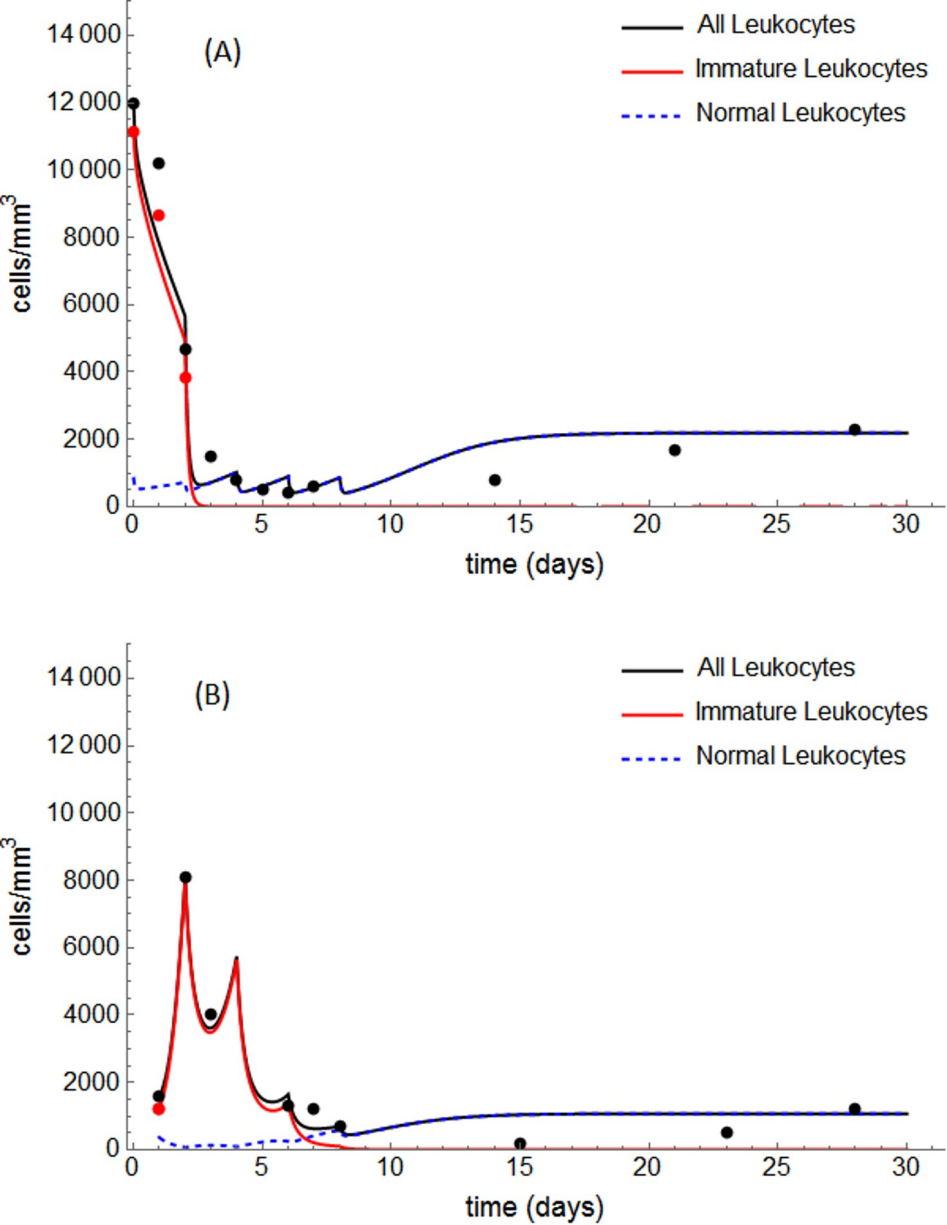
Solid lines represent the solution provided by the adjusted model, whereas dots represent clinical data from patients #18 (A) and #22 (B). The patients received standard ICAL induction protocol (75 mg ATRA every day and 60 mg/day/m^2^ chemotherapy on days 2, 4, 6, 8).

The values obtained for the fitted parameters for two representative patients (#18 and #22) are presented in Table 3. The fitted model solutions are shown in Fig 5. For both patients, the estimated parameter values led to Scenario II. Because of the scarcity of data on immature leukocytes during the induction phase for the other patients in our cohort, we only present results from the application of the proposed method to these two patients.

For each patient, the other 99 final tuples resulting from the estimation method also produce reasonable fits (see S3 Fig). In general, the parameter values of such tuples are close to the estimated values in Table 3, which provide the minimum error. Moreover, we calculated how many of these tuples correspond to the same configuration in the phase portrait produced by the estimated values, which was Scenario II for both patients (see S1 and S2 Figs). For patient #18, 79% of the 100 final tuples correspond to Scenario II, 14% correspond to Scenario III, and 7% correspond to Scenario I. For patient #22, 41% correspond to Scenario II and the other 59% correspond to Scenario III.

### Simulating alternative treatment protocols for induction treatment and assessing cytotoxicity and time for remission

We simulated the use of alternative induction treatment schedules and compared the results with the standard induction protocol proposed by ICAL. In this way, we aimed to investigate the feasibility of less cytotoxic or more convenient protocols for use in clinical practice. The standard ICAL induction protocol corresponds to chemotherapy of 60 mg/m^2^ on days 2, 4, 6, and 8 and ATRA from days 0–30. By changing the chemotherapy dose intensities or days of administration, we simulated alternative, less cytotoxic protocols. The doses were decreased to 30 mg/m^2^ and the days of chemotherapy and intervals between doses were varied. We also simulated alternative protocols with the same chemotherapy schedule as the ICAL protocol, but different ATRA regimens. The simulated alternative protocols are presented in Table 4.

**Table 4:** Assessment of cytotoxicity and time for remission through the simulation of different protocols for patients #18 and #22.

Different Chemotherapy Protocols (plus ATRA from day 0–30)	Patient #18		Patient #22	
	*Nmin* (cells/mm^3^)	*t*_*REM*_ (days)	*Nmin* (cells/mm^3^)	*t*_*REM*_ (days)
P0: CHEMO 60 mg/m^2^ at days 2, 4, 6, 8 (ICAL standard induction protocol)	420	2.9	78	9
P1: CHEMO 30 mg/m^2^ at days 2, 3, 4, 5, 6, 7, 8, 9	510	3.3	72	10.2
P2: CHEMO 60 mg/m^2^ at days 2, 6	468	2.9	50	∞
P3: CHEMO 60 mg/m^2^ at days 2, 4	525	2.9	50	∞
P4: CHEMO 30 mg/m^2^ at days 2, 3, 4, 5	525	3.3	50	∞
P5: CHEMO 30 mg/m^2^ at days 2, 4, 6, 8	525	3.7	50	∞
P6: CHEMO 60 mg/m^2^ at day 2	525	2.9	50	∞
P7: CHEMO 30 mg/m^2^ at day 2	525	3.7	50	∞
P8: no CHEMO (ATRA only)	525	4.9	50	∞
Different ATRA Protocols (CHEMO 60 mg/m^2^ at days 2, 4, 6, 8 (ICAL Induction))	*Nmin* (cells/mm^3^)	*t*_*REM*_ (days)	*Nmin* (cells/mm^3^)	*t*_*REM*_ (days)
Q1: ATRA from day 0–15, with ICAL standard daily dose	420	2.9	78	9
Q2: ATRA from day 0–30, with half of ICAL standard daily dose	420	3	62	11
Q3: ATRA from day 0–15, with twice the ICAL standard daily dose	420	2.4	112	8
Q4: No ATRA (CHEMO only)	220	3.2	22	∞

To measure the cytotoxicity of a given treatment protocol, we define *Nmin* as the lowest level of normal leukocytes in peripheral blood (cells/mm^3^) during induction treatment, as predicted by the model, when simulating different protocol approaches. Thus, we define *Nmin* = min{*N*(*t*),0≤*t*≤30}. Lower values of *Nmin* correspond to more cytotoxic protocols, because more normal leukocytes die, leading to higher risks of complications. Therefore, it is our objective to maximize *Nmin*.

To assess the time until remission in the different protocols, we defined *t*_*REM*_ as the time at which the simulated number of leukemic leukocytes dropped below 10 cells/mm^3^. The lower the value of *t*_*REM*_, the faster that protocol achieves remission. The results for the alternative protocols are presented in Table 4 for patients #18 and #22.

**Fig 6:**
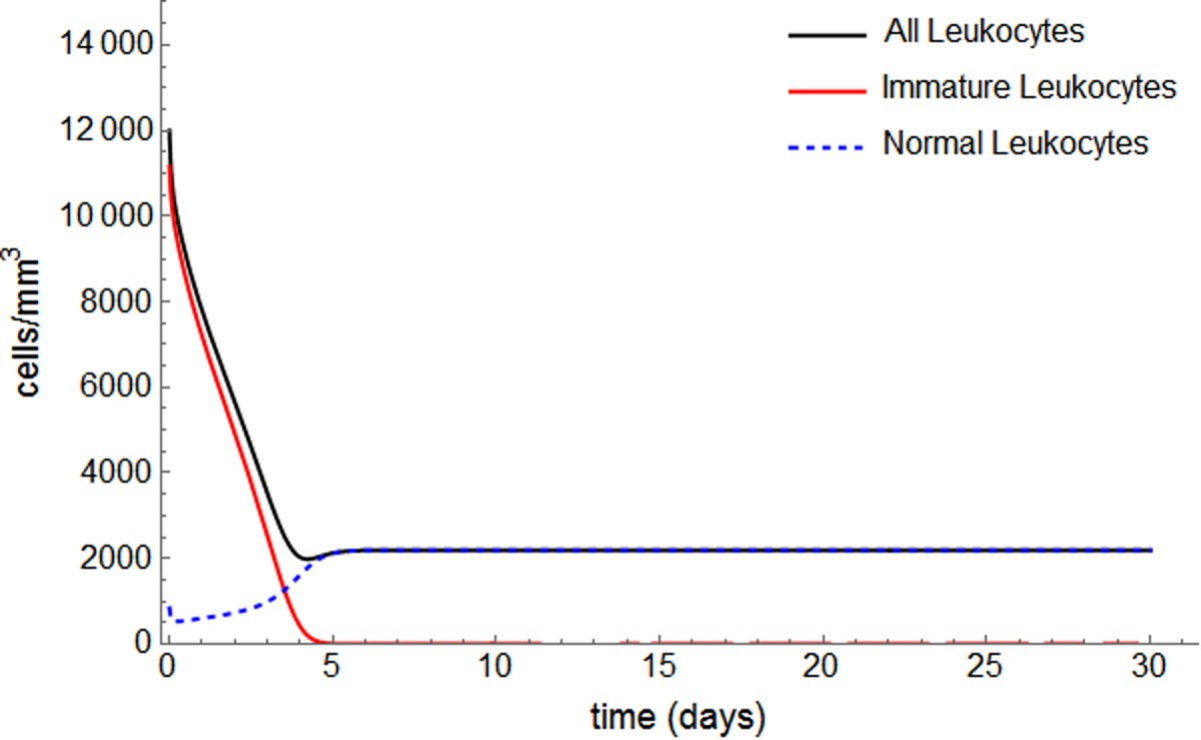
Complete remission achieved with ATRA as single induction agent for patient #18.

For patient #18, all alternative protocols lead to remission. Moreover, it is possible to achieve remission as fast as with the ICAL induction protocol (P1), but with less toxicity. This is achieved by keeping the daily dose of chemotherapy and decreasing the number of doses (P3 and P6). By maintaining the cumulative total dose while increasing the number of doses, the hematological toxicity to normal cells decreases (compare *Nmin* for protocols P0 and P1, for example), because the individual dose amount decreases. Further, remission is even possible using ATRA only, i.e., without the use of cytotoxic chemotherapy (Fig 6). The estimated value for the ATRA effect, *β*_3_ = 0.289005, is sufficiently high to guarantee remission, although more time is required than under the cytotoxic protocols.

For patient #18, we calculated a threshold value of *β*_3_ in which remission is achieved within 30 days using ATRA as a single agent: $\beta_{3}^{th}=0.1658$. If we assume a linear dose-response between ATRA dose and the ATRA effect, this threshold value corresponds to 57% of the ATRA standard dose. When *β*_3_ takes values above this threshold, ATRA alone causes the solutions to migrate from one basin of attraction to another. No randomized clinical trial has offered ATRA as a single agent in induction therapy. Nonetheless, the effect of ATRA in inducing remission is comparable to that of cytotoxic agents. ATRA dosage and the patient's sensibility to the drug influences the value of *β*_3_. Depending on that sensibility, the ATRA dosage may be optimized to achieve remission with minor toxicity, with or without chemotherapy [32,33].

Regarding the protocols in which the ATRA dose is changed, Q1 indicates that the effect of ATRA is relevant in inducing remission in the first 15 days. After that, ATRA can be suspended without any impact on *Nmin* or *t*_*REM*_. In Q3, concentrating the 30-day ATRA dose in only 15 days achieves faster remission without increasing cytotoxicity. Q4 reinforces the importance of ATRA in the induction protocol: without ATRA, toxicity and time to remission are worse.

**Fig 7:**
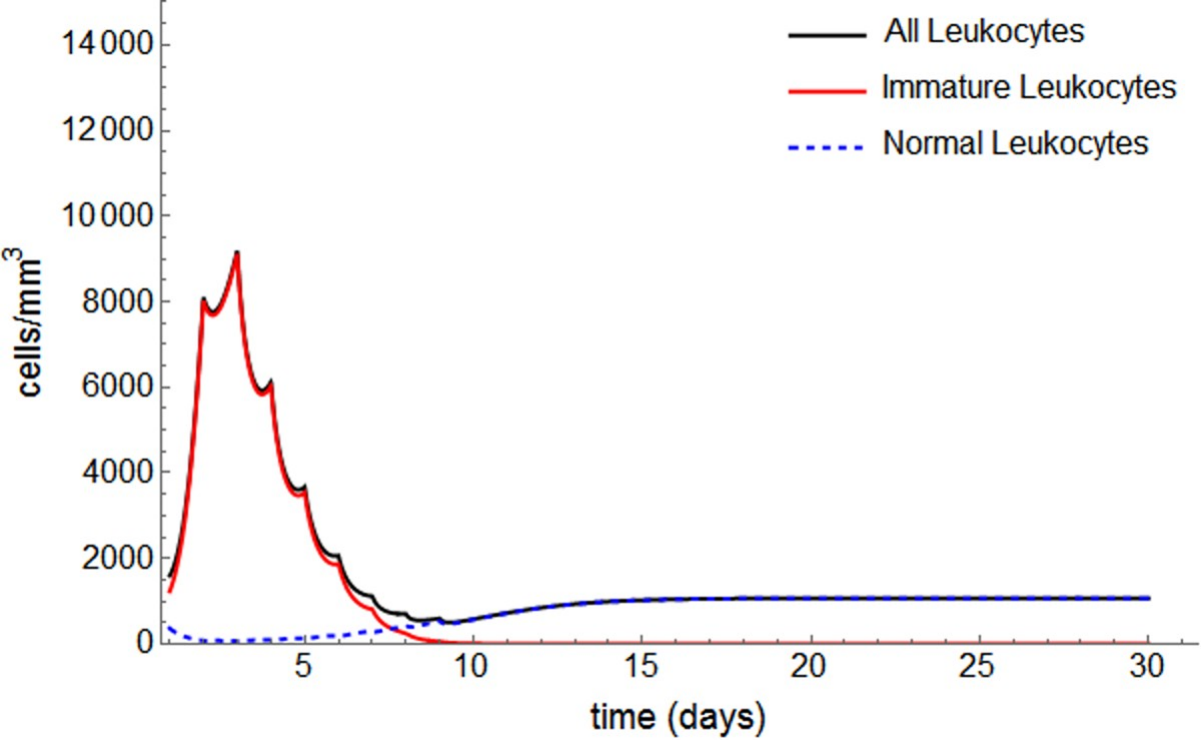
Protocol P1 applied to patient #22.

**Fig 8:**
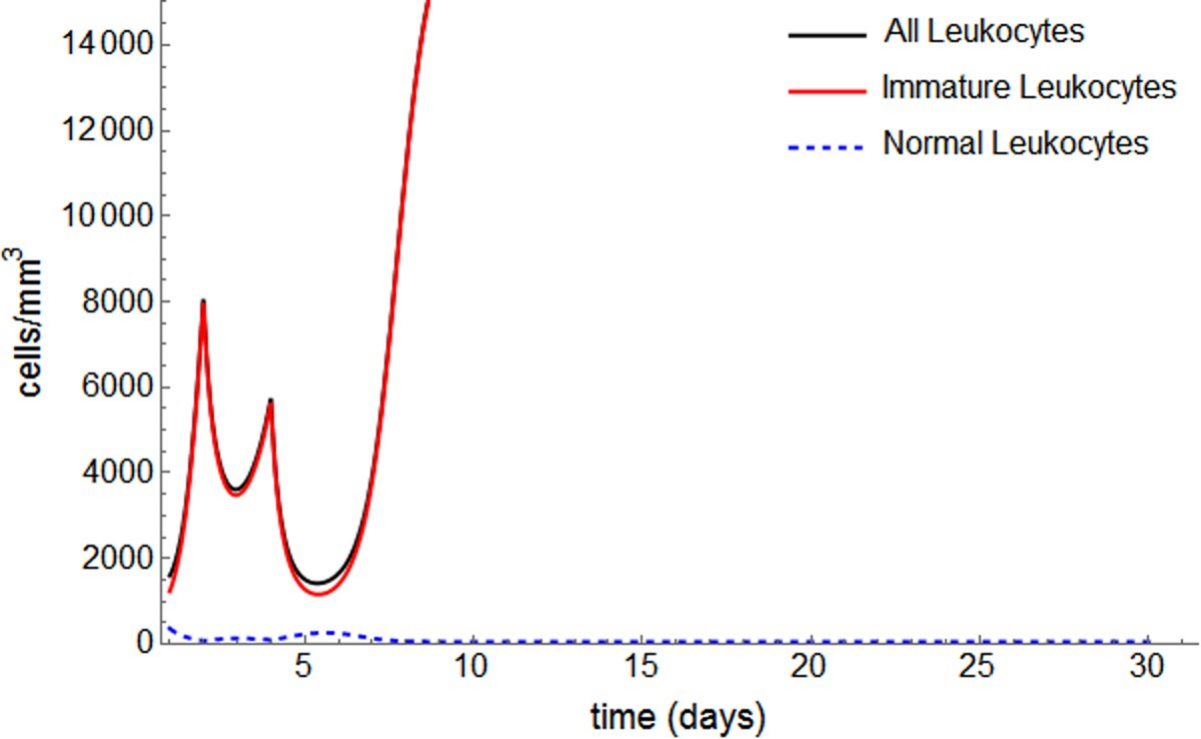
Protocol P3 applied to patient #22.

For patient #22, with the exception of alternative protocol P1, none of the alternative protocols is strong enough to induce remission, and relapse is observed within the first 30 days of induction (Figs 7 and 8).

We can infer the importance of not only the accumulative dose of chemotherapy, but also how it is offered to the patient. For this patient, contrary to #18, P1 is the optimum (faster and less toxic) and only schedule that leads to remission, reinforcing the relevance of risk-adjusted treatment for APL patients.

## Discussion

### Comparing clinical results

The clinical data retrieved from APL patients treated in HCRP reveal very similar population characteristics in comparison with populations from larger clinical trials involving APL patients [4,13]. We have compared our results to other published trials covering APL, IC-APL [4], and Leucemia Promielocitica Aguda 2005 (LPA2005) reported by the Programa Español de Tratamiento en Hematologia/Dutch-Belgian Hemato-Oncology Cooperative Group (PETHEMA/HOVON) [13], as presented in Table 5.

**Table 5:** Comparison between trials.

Characteristic	HCRP–n (%)	IC-APL–n (%)	LPA2005 –n (%)
Male	20 (53)	88 (48)	209 (52)
Female	18 (47)	95 (52)	193 (48)
Bleeding	14 (37)	114 (62)	-
Remission	33 (87)	153 (80)	372 (93)
Death	9 (24)	37 (20)	34 (9)
Death during induction	4 (11)	27 (15)	30 (7)
Relapse	6 (16)	9 (5)	21 (5)
Initial relapse risk–criteria PETHEMA/GIMEMA			
High	9 (24)	58 (32)	118 (19)
Intermediate	26 (68)	95 (52)	200 (50)
Low	3 (8)	30 (16)	84 (21)

We can observe some similarities among the populations considered in these studies. Men and women are equally affected by the disease. The death rate during induction is low, and most patients are classified as having a low or intermediate PETHEMA/GIMEMA risk of relapse. Common toxicities are asymptomatic cardiotoxicity and febrile neutropenia. In contrast to previous reports, our data cover a longer follow-up period (41.8 versus 28 and 38 months for IC-APL and LPA2005, respectively). OS at two years (78.7%) is comparable to that of IC-APL (79.4%), but lower than for LPA2005 (91.5%). The two-year RFS is 78.7%; IC-APL and LPA2005 reported RFS rates of 89.8% and 90.7%, respectively.

### Models in APL

The rarity of APL and well-known pathogenesis depending on a specific cytogenetic rearrangement make it a good candidate for *in vitro* models, e.g., HL-60, NB-4, and PL-21 cell lines [34]. Recent results on diagnosis and treatment response still rely on *in vitro* models [35,36]; animal models are less common, usually reserved for drug toxicity evaluations [37,38].

Many disease and treatment models for acute leukemia based on dynamical systems have already been proposed, but translating their predictions into clinical practice is challenging. In a series of articles, Rubinow and Lebowitz established an ecological model for the pathology. Starting from normal WBC production, they developed a model in which normal and leukemic cells compete in compartmental environments, eventually outputting the effect of chemotherapy in AML treatment [19,30,39]. Afenya proposed a population-based model in which healthy cells and leukemic cells compete for resources in the same environment, and performed numerical simulations both with and without the influence of cytotoxic chemotherapy [20]. His model predicted that high doses of cytotoxic drugs over short timescales were feasible and could lead to long-term relapse-free conditions. Afenya and colleagues then extended these results, including other biological aspects of the disease that would impact in the dynamical behavior, such as diffusion [40]. However, APL has a biological behavior that is distinct from other AMLs, which has implications for treatment design. Hence, a model that incorporates APL characteristics is preferable.

Based on the North American Intergroup trial INT0129 trial protocols, Werner and colleagues proposed an APL multicompartment model of hematopoiesis and estimated the average duration of induction therapy for the leukemic stem-like cells to be eradicated [41]. However, the leukemic burden is assessed by evaluating the number of copies of PML-RARα transcripts, which demands real-time polymerase chain reaction analysis. This may not be available at every care center. In a multicompartmental approach, the dynamics in the bone marrow play a fundamental role. This aspect compromises the applicability of this approach in medical routines, as numerous bone marrow aspirates are required. Our model depends only on peripheral blood test results to provide predictions, making it relatively easy to incorporate into clinical practice.

### Model fitting and prediction capability

The scarcity of information on leukemic cell populations is because, in the majority of samples, a simple daily blood test (WBC count and proportion of lymphocytes) was run during the induction phase. Of the 38 patients in the cohort, all presented at least one value for WBC in the first 30 days after diagnosis, with a median of 11 values for this variable. In terms of the promyelocyte count, 26 patients (68%) had this information available, with a median of only one value. Note that small variations in *β*_1_ and *β*_2_ in the neighborhood of the threshold values separating Scenarios II and III lead the solutions to completely different long-term outcomes. For this reason, more information on the promyelocyte population (produced by a daily complete blood count, for example) is fundamental for determining parameter values that ensure the models fit the clinical data well. This should result in more reliable long-term predictions, for example, to classify a patient at high risk of relapse if a global minimum is found in the subset of Scenario III. However, for such predictions, the effect of consolidation, maintenance, and parameter variations over time may influence the outcome.

Despite this limitation on long-time predictions, one must recognize that the objective of a risk-prediction method is to provide information about the probability of occurrence of an event *a priori*, so that an intervention can modify this probability. Most of the prognostic tools for many oncologic pathologies only consider diagnosis information in anticipating how the disease will evolve. The PETHEMA/GIMEMA criterion itself was developed in this way. When applied to our cohort, it returns a Negative Predictive Value (NPV) of 82.8% for relapse in high-risk patients. When only the initial WBC count is considered, as suggested by the French-Belgian-Swiss APL group [42], the NPV drops to 80%. As APL treatment is based on the risk of relapse, a high NPV is beneficial, providing less-intensive therapy for lower-risk patients.

The lack of consistency in the predictive power of prognostic factors and tools can be attributed to the APL epidemiology, which provides little information for adequate population-based regressive models. For example, the presence of a mutation on gene FLT3 (FLT3-IDT) was presumed to be associated with poorer prognosis, but several studies showed different results, meaning the effect of the mutation on treatment choice remains undetermined [43–47].

Incorporating the impact of the treatment into the risk assessment is also relevant. PREDICT is a population-based prognostic model for breast cancer that considers patient and tumor characteristics and the treatment to be offered, helping the physician select the optimum treatment protocol [48]. The PREDICT tool relies on a retrospective cohort of more than 5,000 patients, validated by data from other studies covering thousands of patients [49]. Once again, the scarcity of data makes such a methodology unfeasible for APL.

For the selected patients (#18 and #22), there is a good fit between the model simulations and the clinical data. Nevertheless, we have obtained pairs of parameter values that produce a good fit to the data, but lead to different scenarios, thereby indicating different long-term outcomes (relapse versus sustained remission, reflected by Scenarios III and II, respectively). In other words, for our cohort, the data collected during the induction therapy are insufficient to allow the estimation method to provide a single pair of parameters (a global minimum), or even a set of local minima corresponding to the same scenario, leading to the same outcomes. We calculated the number of local minima in each scenario. For patient #18, although the local minima are more dispersed about the parameter space, most of them correspond to Scenario II. Therefore, we can say that the estimation for patient #18 is robust with regard to variations in other local minima of *E(X)*. For patient #22, the local minima are more concentrated in certain regions of the parameter space, but are somehow well divided between Scenarios II (59%) and III (41%). Although such results suggest the possibility of different outcomes for patient #22 when other local minima are used as parameter values, they also indicate that this patient is more likely to suffer a relapse than patient #18. This information agrees with our simulation of different protocols, where far fewer protocols lead to remission for patient #22 than for patient #18. Such an evaluation could constitute a method for estimating the risk of relapse.

### Example of an application

For the reasons discussed above, a disease model with no dependency on “big data” [50] that incorporates treatment impacts in an adaptable fashion can be useful in many applications. In this section, we introduce one such application by establishing a method for comparing different induction protocols.

Testing new drugs or treatment schedules demands a considerable amount of time as well as financial and human resources. Releasing new antineoplastic therapies is five times more expensive than other medications, mostly because of the associated research & development costs [51]. In this context, *in silico* experiments optimize the use of resources [52,53]. For APL induction protocols, we present two quantities that can be used to evaluate different schedules. Other quantities of interest can be investigated, e.g., costs with drug administration. These quantities can be arranged in a cost function to be minimized, or a maximum value for the cost function can be set, and any schedule that surpasses this value is removed from the clinical trial. For example, in our simulations, protocols with 30 mg/m^2^/day of anthracycline are not attractive because 60 mg/m^2^/day leads to remission in less time with the same toxicity. It is possible to include other cytotoxic medications in the simulations by adding suitable terms and adapting the existing set of equations. Contemplating new treatment modalities, such as immunotherapy, would require a complete change of paradigm.

Comparing the fitted values for the parameters of patients #18 and #22 in Table 3, we notice that patient #18 has a more intense production of normal cells (*r*_*N*_), which contribute to a stronger control on leukemic cells. Additionally, the aggressiveness of leukemic cells (reflected by parameter *β*_1_) is four times smaller in patient #18. The aggressiveness of normal cells (*β*_2_) is similar in both patients. Finally, the effects of treatment are stronger in patient #18 (both ATRA and chemotherapy, described by higher values of *β*_3_ and *γ*, respectively), indicating that he would respond better to the induction treatment than patient #22, including remission with ATRA only, as reported by Warrell and colleagues [54]. Such evaluation of aggressiveness may be used to stratify the risk of relapse, where patient #22 is at a higher risk than patient #18, although the implications on clinical practice are yet to be determined.

## Conclusion

The analyzed subset of Brazilian patients subjected to the ICAL protocol presented similar characteristics as other published cohorts, with a longer follow-up and a higher relapse rate. The proposed mathematical model captures the dynamics of leukemic and normal leukocytes in peripheral blood provided by simple laboratory tests. The information produced by the model may be useful in determining the risk of relapse and in the development of novel treatment protocols, as clinical trials on APL are challenging due to the scarcity of patients, although the quality and quantity of data play a fundamental role in the power of prediction.

## Supporting information

S1 Fig Histograms (in the diagonal) and scatter plots showing the distributions of the 100 local minima of E(X) that result from the parameter estimation method for patient #18. In the scatter plots, the color scale corresponds to the values of E(X), with blue for low values and red for high values. The parameter tuple that produces the lowest value of E(X), and corresponds to the estimated value in Table 3, is indicated by the symbol “+” with cyan color.
(TIF)

S2 Fig Histograms (in the diagonal) and scatter plots showing the distributions of the 100 local minima of E(X) that result from the parameter estimation method for patient #22. In the scatter plots, the color scale corresponds to the values of E(X), with blue for low values and red for high values. The parameter tuple that produces the lowest value of E(X), and corresponds to the estimated value in Table 3, is indicated by the symbol “+” with cyan color.
(TIF)

S3 Fig Model simulations using the 100 local minima of E(X) that result from the parameter estimation method for patient #18 (A) and patient #22 (B).
(TIF)
