# Supplementary figures and images for: Modeling dynamics and alternative treatment strategies in acute promyelocytic leukemia
Source: PLoS One. 2019 Aug 15;14(8):e0221011. doi: 10.1371/journal.pone.0221011 (PMC6695187; doi:10.1371/journal.pone.0221011)

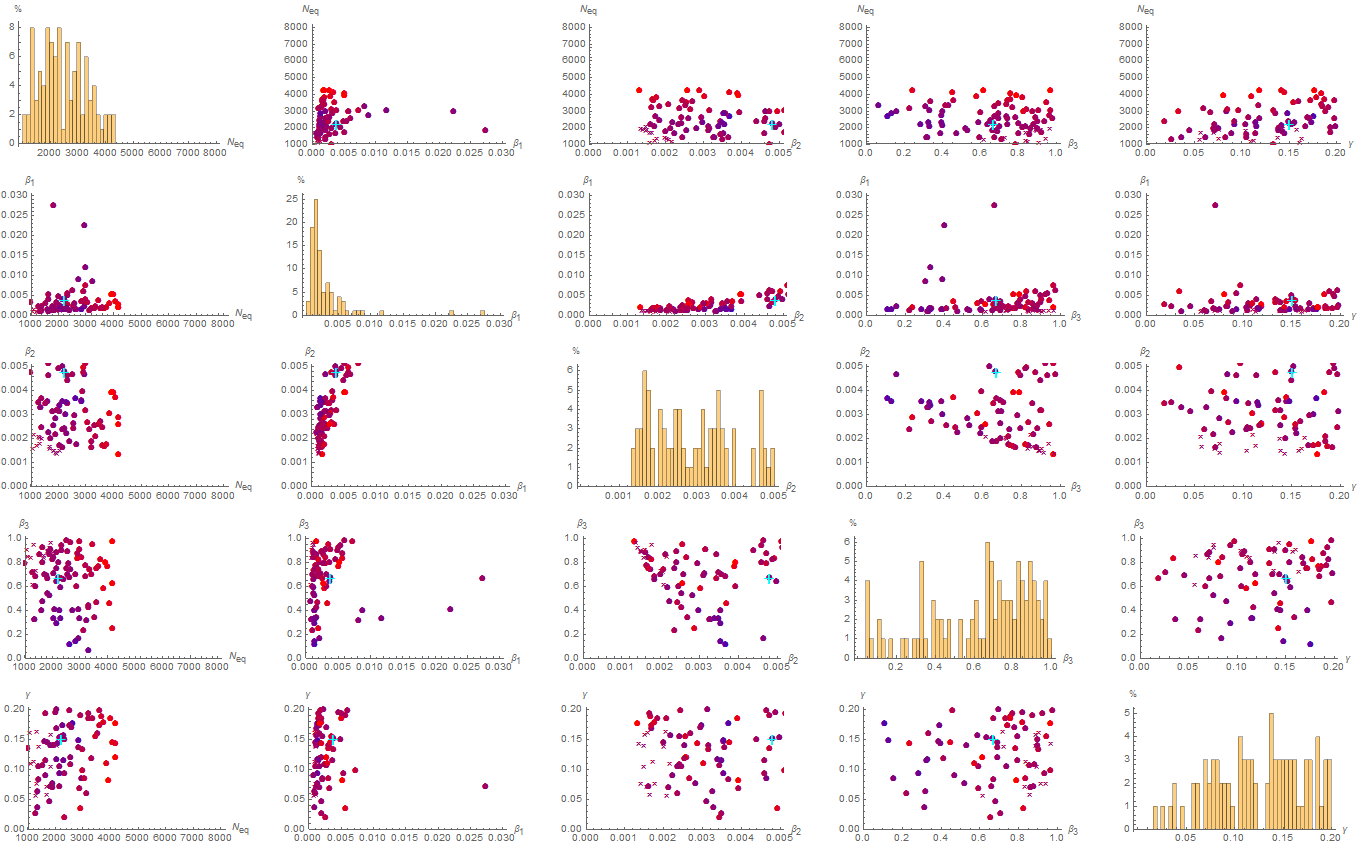

Supplement: S1 Fig — The parameter tuple that produces the lowest value of E(X), and corresponds to the estimated value in Table 3, is indicated by the symbol “+” with cyan color. (TIF) [file pone.0221011.s001.tif]

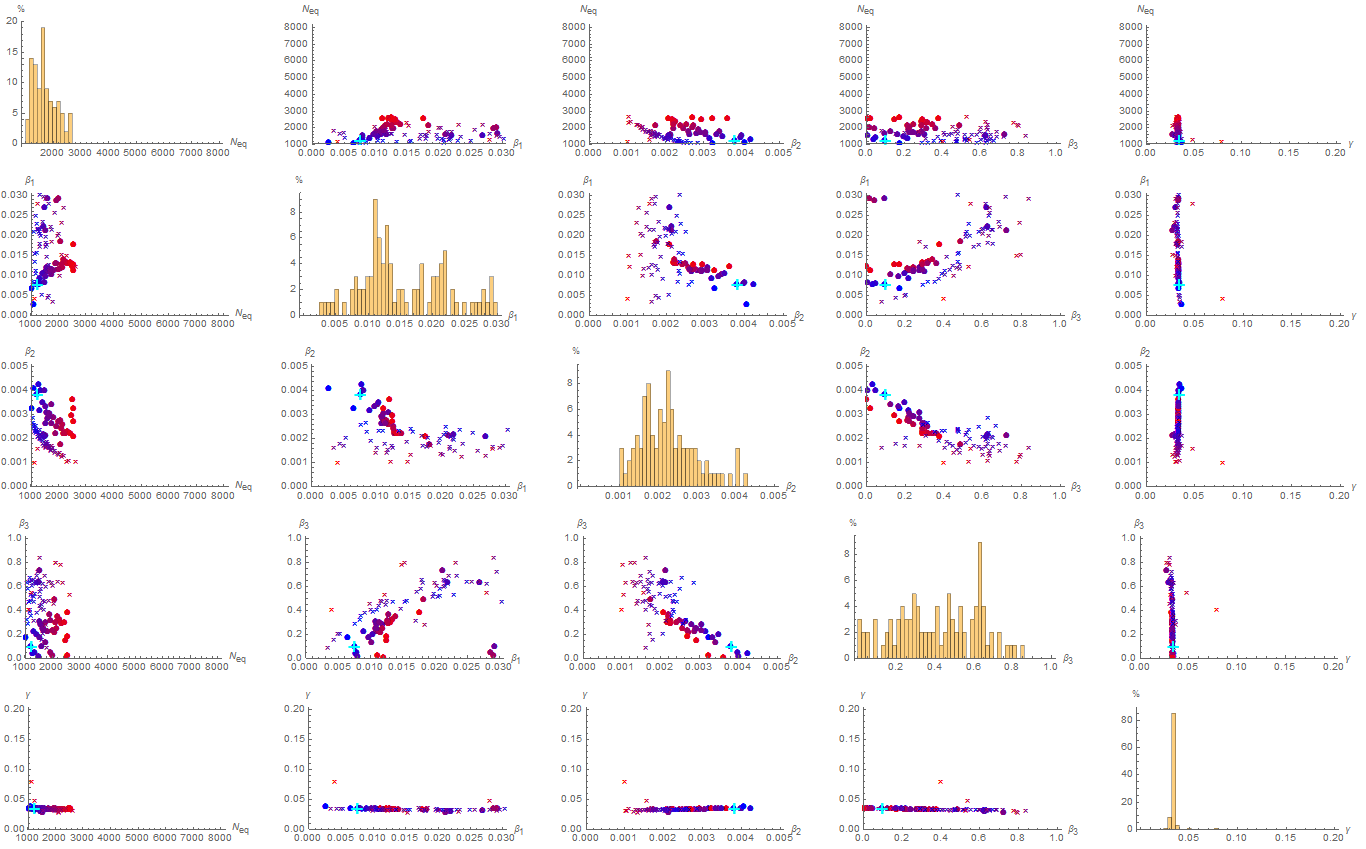

Supplement: S2 Fig — In the scatter plots, the color scale corresponds to the values of E(X), with blue for low values and red for high values. The parameter tuple that produces the lowest value of E(X), and corresponds to the estimated value in Table 3, is indicated by the symbol “+” with cyan color. (TIF) [file pone.0221011.s002.tif]

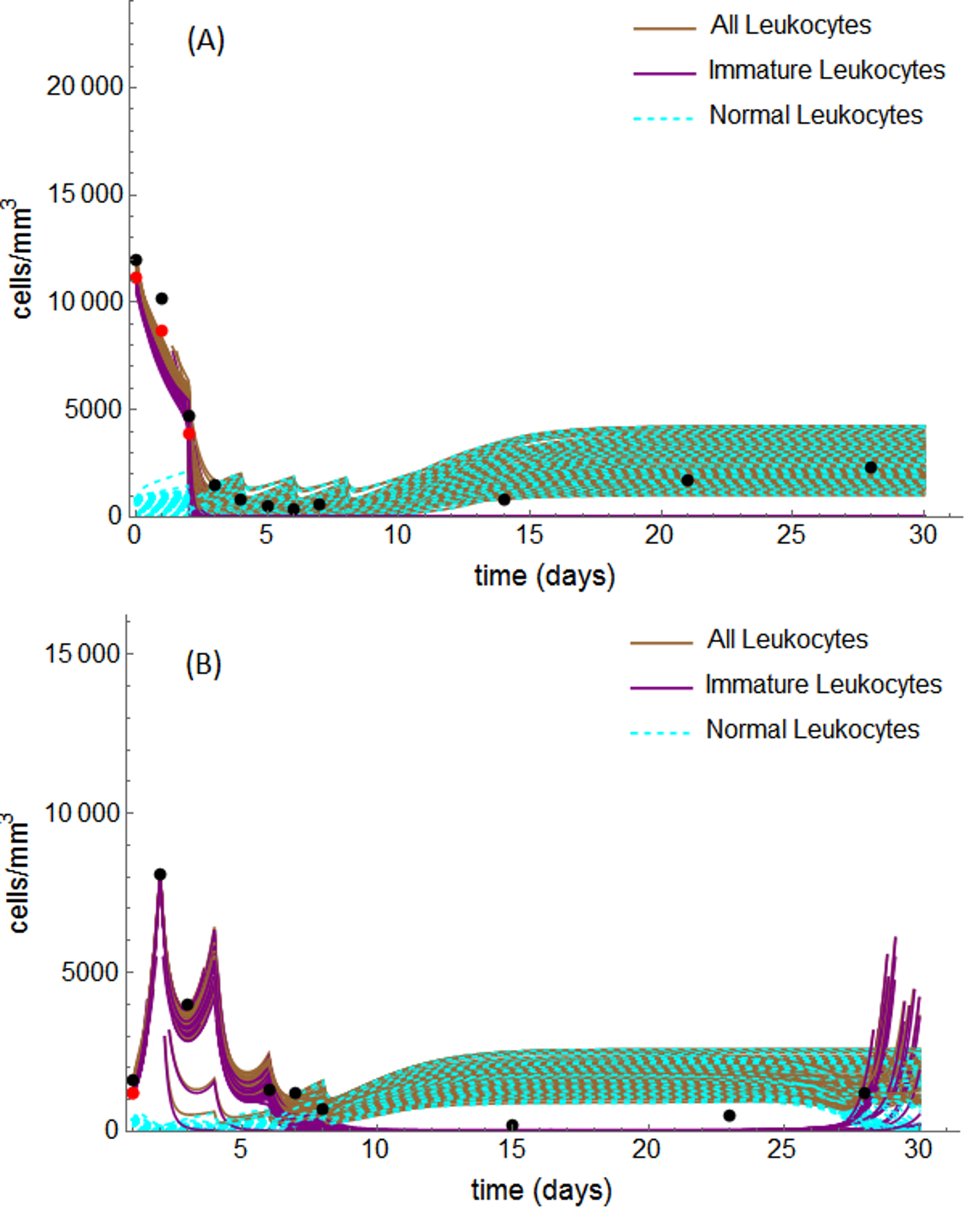

Supplement: S3 Fig — Model simulations using the 100 local minima of E(X) that result from the parameter estimation method for patient #18 (A) and patient #22 (B). (TIF) [file pone.0221011.s003.tif]
